# Supplementary material for: Losartan as a mechanotherapeutic adjuvant: Remodeling the breast tumor microenvironment to improve treatment efficacy
Source: PLoS One. 2025 Jul 11;20(7):e0328196. doi: 10.1371/journal.pone.0328196 (PMC12250197; doi:10.1371/journal.pone.0328196)
Supplement: S1 File — These include the full set of governing equations (S1–S13 Equations), detailed model assumptions, boundary and initial conditions, and parameter values used in the simulations (S1–S6 Tables). The supplementary document also elaborates on losartan pharmacodynamics. S1 Table. Biological parameters (tumor cell kinetics and oxygen consumption). S2 Table. Transport properties (fluid and molecular transport). S3 Table. Losartan PK/PD parameters. S4 Table. Mechanical properties of tissue. S5 Table. Vascular filtration parameters (Starling’s law). S6 Table. Initial conditions in the simulations (pretreatment). (DOCX) [file pone.0328196.s001.docx]

**Supplementary material**

This supplementary document provides additional details of the computational model, including the full governing equations, parameter values (with tables), boundary and initial conditions, and further notes on model assumptions and implementation. Equations and tables here are labeled with an “S” (for Supplementary) for clarity.

# S1. Governing equations of the model

1. **Tumor cell population dynamics:** The spatio-temporal evolution of cancer cell density n (cells/mm³) is described by a reaction–diffusion equation with source and sink terms:

- **(S1) (governing equation for n)**

$$\frac{\partial n}{\partial t}=\nabla\cdot\left( D_{\mathrm{cell}}\nabla n \right)+S-T$$

Here D_cell_ is the effective diffusion coefficient of tumor cells (random motility). S represents the proliferation source term (cells produced per unit volume per unit time), and T represents the loss term due to treatment-induced cell death. In our model, S and T are functions of local drug concentration C_drug_ as well as cell density and time.

- **(S2) Gompertzian proliferation:**

Tumor cell proliferation follows Gompertz law:

$$S=\rho n\mathrm{Ln} \left( \frac{n_{max}}{n} \right)$$

where ρ is the nominal proliferation rate (1/s) and n_max_ is the carrying capacity (maximum cell density). This form causes growth to slow as n approaches n_max_. The parameters ρ and n_max_ are given in Table S1.

1. **Drug-modified proliferation and death:** Losartan’s effects enter through α and β factors that modulate proliferation and death, respectively. We model these via sigmoidal dose–response functions:

- **(S3) (proliferation inhibition factor**)

$$\alpha=\alpha_{\max}\frac{C_{drug}}{C_{drug}+C_{50}}$$

**(S4)** **(apoptosis induction factor)**

$$\beta=\beta_{\max}\frac{C_{drug}}{C_{drug}+C_{50}}$$

where α_max_ is the maximum fractional reduction in proliferation rate, β_max_ is the maximum induced fractional death rate, and C_50_ is the concentration at which half of the maximal effect is achieved. (For simplicity, we used the same C_50_ for both processes, as shown in Table S3.) Using these, we define the sink in Eq. (S1) as:

$$T=\left( \alpha+\beta\right)n$$

In other words, losartan reduces the proliferation rate by a factor proportional to α and adds an apoptosis term proportional to β. At C_drug_ = 0, α = 0, β = 0, so we recover standard Gompertz growth. At high drug concentrations, proliferation can be heavily suppressed and apoptosis greatly enhanced.

1. **Oxygen transport:** Oxygen concentration C_O2_ [mol/mm³] in tissue is governed by convection–diffusion with sources and sinks:

- **(S5)** **(oxygen transport equation)**

$$\frac{\partial\left( \phi_{s}C_{O_{2}} \right)}{\partial t}+\nabla\left( \boldsymbol{v}_{f}C_{O_{2}} \right)=\nabla\cdot\left( D_{O_{2}}\nabla C_{O_{2}} \right)+r-\phi n$$

Here *D*_O2_ is the diffusion coefficient of oxygen in tissue (mm²/s), and *v_f_* is the interstitial fluid velocity (mm/s) which convects oxygen. The term *r* is the oxygen supply rate from blood vessels (mol/mm³/s), and *ϕ* is the oxygen consumption rate per cell (mol/(cell·s)). The product **ϕ⋅n** gives oxygen consumption per unit volume.

The oxygen supply term is active in regions with perfused vessels. We model it as:

- **(S6) (oxygen supply term equation)**

$$r=P_{O_{2}}S_{v}\left( \left( C_{O_{2}} \right)_{v}-C_{O_{2}} \right)$$

where P_O2_ is the effective permeability of oxygen across capillary walls (mm/s), S_v_ is the surface area of capillaries per tissue volume (mm²/mm³), and (C_O2_)_v_ is the dissolved oxygen concentration in blood. This form supplies oxygen at a rate proportional to the concentration difference between blood and tissue. Table S2 lists the values used, which yield realistic oxygen levels in well-perfused tissue.

1. **Drug pharmacokinetics (PD):** We explicitly model losartan parent drug and metabolite concentrations in the tissue, C_parent_ and C_metabolite_ (mol/m³), assumed spatially uniform (well-mixed in tissue via blood perfusion):

- **(S7) (rate equation for C_parent_)**

$$C_{\mathrm{parent}}=\left( {f_{\mathrm{breast}}f}_{\mathrm{bio}}d_{\mathrm{mol}}/V_{\mathrm{breast}} \right)e^{-\left( k_{\mathrm{clearance}} \right)t}$$

- **(S8) (rate equation for C_metabolite_)**

$$C_{\mathrm{metabolite}}=\left( {f_{\mathrm{breast}}f}_{\mathrm{metabolite}}d_{\mathrm{mol}}/V_{\mathrm{breast}} \right)e^{-\left( k_{\mathrm{metabolite}} \right)t}$$

Equation (S7) states that the parent losartan concentration decays with first-order rate constant *k*_clearance_ = Ln 2/t_1/2_ (s⁻¹), consistent with losartan’s elimination half-life (~2 h). Equation (S8) states that the active metabolite EXP3174 concentration decays with first-order rate constant *k*_metabolite_ = Ln 2/t_1/2_ (half-life ~6–9 h). Here *d*_mol_ is the administered dose in moles (50 mg losartan = 0.1183 mmol), *f*_breast_ is the fraction of cardiac output to breast tissue (~5%), *f*_bio_ is bioavailability (33%), *f*_metabolite_ is the fraction of eliminated losartan converted to metabolite based on pharmacokinetic data (Table S3), and V_breast_ is the perfused breast tissue volume in the model. The total tissue drug concentration used in the PD functions is C_drug_ = C_parent_ + C_metabolite_.

1. **Solid mechanics (poroelasticity):** The solid deformation satisfies force equilibrium (neglecting inertia):

- **(S9) (mechanical equilibrium equation)**

$$\nabla\cdot\boldsymbol{\sigma}=\mathbf{0}$$

where **σ** is the Cauchy stress tensor (Pa). We use a finite-strain neo-Hookean hyperelastic model for the stress–strain behavior. In terms of the second Piola–Kirchhoff stress **S** and Green–Lagrange strain **E**:

- **(S10) (constitutive relation equation)**

$$S=\frac{\partial W}{\partial E}$$

where *W* is the strain energy density for a compressible neo-Hookean material. We take

$$W=\phi_{s}\left( \frac{1}{2}\mu\left( I_{1}-3 \right)-\mu\ln\left( J_{el} \right)+\frac{1}{2}\lambda\left[ \ln\left( J_{el} \right) \right]^{2} \right)$$

with Lamé parameters λ, μ (see Table S4), J_el_ = det (**F**) the elastic volume ratio (Jacobian), and I_1_ = tr (**C**) the first invariant of the right Cauchy–Green tensor **C** = **F**^T^**F**. This yields the standard neo-Hookean constitutive law. The tumor and normal tissues have different λ, μ values (reflecting their different moduli).

1. **Mass conservation of solid phase:** Tumor growth adds mass to the solid phase (cells), causing tissue expansion. The solid mass balance is:

- **(S11) (solid mass balance equation)**

$$\frac{\partial\left( \rho_{s}\phi_{s} \right)}{\partial t}+\nabla\cdot\left( \phi_{s}\rho_{s}\boldsymbol{v}_{s} \right)=m_{\mathrm{cell}}S$$

*ρ*_s_ is solid density (kg/mm³), *ϕ*_s_ is solid volume fraction, and ***v*_s_** is the solid (matrix) velocity. The source term on the right adds solid mass due to cell proliferation, where m_cell_ is the mass of one cell (~10^-9^ kg) and S is the proliferation rate (Gompertz form from S2). In implementation, this causes volumetric growth of the solid. If losartan inhibits proliferation (α), the solid source is reduced accordingly (less growth). Similarly, if T > S (more death than growth), the solid content effectively decreases (tissue can contract)

1. **Mass conservation of fluid phase:** Interstitial fluid flow in the tumor microenvironment is described by Darcy’s law and fluid continuity:

- **(S12) (fluid mass balance equation)**

$$\frac{\partial\left( \rho_{f}\phi_{f} \right)}{\partial t}+\nabla\cdot\left( \phi_{f}\rho_{f}\boldsymbol{v}_{f} \right)=\rho_{f}Q_{f}$$

*ρ*_f_ is fluid density, *ϕ*_f_ is fluid volume fraction (porosity), ***v*_f_** is interstitial fluid velocity, and *Q*_f_ is the net fluid source from blood/lymph. We define ***v*_f_** by Darcy’s law: ***v*_f_** ​= −κ∇p_i_​, where κ is hydraulic conductivity (m^3^·s/kg) and p_i_ is interstitial fluid pressure. Distinct κ values were used for tumor and normal tissue (Table S2).

The source term *Q*_f_ is given by Starling’s principle for fluid exchange:

- **(S13) (Starling’s fluid source equation)**

$$Q_{f}=\Theta_{c}\left[ \left( p_{c}-p_{i} \right)-\bar{\sigma}\left( \pi_{c}-\pi_{i} \right) \right]-\Theta_{l}\left( p_{i}-p_{l} \right)$$

Here *p*_c_ is capillary blood pressure, *p*_l_ is lymphatic pressure, *π*_c_ and *π*_l_ are plasma and interstitial oncotic pressures, respectively. *Θ*_c_ and *Θ*_l_ are the capillary and lymphatic filtration coefficients (s⁻¹), and σ̄ is the osmotic reflection coefficient. The first term *Θ*_c_(*p*_c_ – *p*_i_) represents fluid filtration out of blood capillaries, the second term *Θ*_l_(*p*_i_ – *p*_l_) represents fluid drainage into lymphatics, and the last term σ̄ (*π*_c_ – *π*_l_) represents the opposing effect of osmotic pressure difference (effective suction of fluid back into capillaries). Parameter values are in Table S5. In our baseline, tumor tissue has high *p*_i_ (12 mmHg) and essentially no lymphatics (*Θ*_l_ ​≈ 0), leading to net outward fluid flow that raises IFP.

1. **Summary:** The coupled equations (S1)–(S13) form the core of our model. They were solved simultaneously at each time step to capture the interactions between tumor cell dynamics, oxygenation, drug distribution, and tissue mechanics. The next sections list the parameter values and discuss boundary/initial conditions and additional notes on model assumptions.

# S2. Model parameters

Tables S1–S6 list all parameter values used in the simulations, organized by category. References are provided for values from literature. When direct data were unavailable, parameters were estimated or calibrated as noted.

Table S1: Biological parameters (tumor cell kinetics and oxygen consumption).

| parameter | Description | Tissue | Value | Ref |
| --- | --- | --- | --- | --- |
| Φ (mol/cell/s) | Cellular O₂ consumption rate | Healthy  Tumor | 20.8×10^-18^  32.5×10^-18^ | [[1](#_ENREF_1)] |
| (C_O2_)_v_ (mol/m^3^) | dissolved O₂ in vessels | Healthy  Tumor | 0.0884  0.0420 | [[2](#_ENREF_2)] [[3](#_ENREF_3)] [[1](#_ENREF_1)] |
| n_max_ (cells/cm^3^) | Cell carrying capacity (tumor) | – | 191,000 | [[4](#_ENREF_4)] |
| ρ (cell/day) | Proliferation rate of cancer cells | Tumor | 1/3.2 | [[5](#_ENREF_5)] |
| m_cell_ (kg) | Mass of one cell | Both | 1.0×10^-9^ | [[6](#_ENREF_6)] (calc.) |

Notes: Tumor cells have a higher oxygen consumption rate than normal cells, reflecting more aggressive metabolism. The carrying capacity n_max_ is based on literature estimates of breast tumor cellularity, representing the cell density of a fully packed tumor. The proliferation rate ρ corresponds to a cell doubling time of about 3.2 days.​

Table S2: Transport properties (fluid and molecular transport).

| parameter | Description | Tissue | Value | Ref |
| --- | --- | --- | --- | --- |
| κ  cm^2^(mmHg s)^-1^ | Hydraulic conductivity (IFP) | Healthy  Tumor | 4.13×10^-8^  8.53×10^-9^ | [[7](#_ENREF_7)] |
| D_o2_(m^2^/s) | O₂ Diffusion coefficient | All | 2.16×10^-9^ | [[8](#_ENREF_8)] |
| D_cell_ (cm^2^/day) | Cell Diffusion | All | 0.0013 | [[8](#_ENREF_8)] |

Notes: Hydraulic conductivity is much lower in tumor tissue (indicating much greater resistance to fluid flow). O₂ diffusivity is assumed equal in both tissues (similar composition). The cell diffusion coefficient is small, representing limited random motility of cancer cells.

Table S3:. Losartan PK/PD parameters

| parameter | Description | Value | Ref |
| --- | --- | --- | --- |
| α_max_ (1/h) | Max proliferation inhibition rate by Losartan | 0.0173 | Calibrated (see text) |
| β_max_ (1/h) | Max apoptosis rate by Losartan | 0.0264 | Calibrated (see text) |
| C_50_ (µM) | Half-max effective concentration | 0.5 | Calibrated (see text) |
| f_bio_ | Oral bioavailability of Losartan | 0.33 | [[9](#_ENREF_9)] (Drug Bank) |
| f_metabolite_ | Fraction of dose Metabolized to EXP3174 | 0.14 | [[9](#_ENREF_9)] (Drug Bank) |
| 𝑓_breast_ ​ | Fraction of cardiac output to breast | 0.05 | [[10](#_ENREF_10)] (PET imaging) |
| t_1/2_ (h) | Losartan half-life | 2 (1.5–2.5) | [[9](#_ENREF_9)] (Drug Bank) |
| t_1/2_ (h) | EXP3174 half-life | 6 (6–9) | [[9](#_ENREF_9)] (Drug Bank) |

Notes: We estimated the pharmacodynamic parameters αₘₐₓ, βₘₐₓ, and C₅₀ based on experimental data from the literature. For αₘₐₓ, 1 μM losartan was reported to reduce prostate cancer cell survival to ~66% over 24 hours [[11](#_ENREF_11)]. Assuming exponential decay of viable cells count $S(t) = S_{0}e^{-\alpha_{\max}t}$ and solving $0.66 = 1.0e^{-\alpha_{\max}\times24}$ yields $\alpha_{\max}=0.0173 h^{-1}$, representing the maximal rate of proliferation inhibition. For βₘₐₓ,10 μM losartan increased apoptosis from 3.7% (at 24 h) to 13.1% (at 72 h) [[12](#_ENREF_12)]. Assuming an exponential increase in apoptosis, we solve $0.131 = 0.037e^{\beta_{\max}\times48}$, giving $\beta_{\max}=0.0264 h^{-1}$, corresponding to the maximal rate of apoptosis induction. We chose C₅₀ = 0.5 μM as the concentration producing ~50% of the maximal effect, consistent with the finding that 1 μM losartan yields ~66% survival (a substantial inhibition at a sub-maximal dose). While these estimates are reasonable, further validation across cancer types and experimental conditions is warranted.

Table 4: Mechanical properties of tissue.

| parameter | Description | Tissue | Value | Ref |
| --- | --- | --- | --- | --- |
| E (kPa) | Elastic modulus (Young) | Healthy  Tumor | 18  42 | [[13](#_ENREF_13)]  [[14](#_ENREF_14)] |
| ν | Poisson's ratio | Both | 0.45 | [[15](#_ENREF_15)] (nearly incompressible) |
| ϕ_b_ | Initial porosity | Healthy  Tumor | 0.644  0.455 | [[16](#_ENREF_16)] |
| ρ_s_ (g/cm^3^) | Solid phase density | Healthy  Tumor | 1.04 | [[17](#_ENREF_17)] |
| ρ_f_ (g/cm^3^) | Interstitial fluid density | - | 1 | [[18](#_ENREF_18)] |
| μ (Pa·s) | Interstitial fluid viscosity | - | 0.0035 | \| [[19](#_ENREF_19)] (~3.5 cP) \| \| --- \| |

*Notes*: Tumor tissue is significantly stiffer (E = 42 kPa) than normal (18 kPa). Both are treated as nearly incompressible (ν = 0.45). Tumor porosity is lower due to high cell/ECM content in the tumor.

Table 5: Vascular filtration parameters (Starling’s law).

| parameter | Description | Tissue | Value | Ref |
| --- | --- | --- | --- | --- |
| L_c_  (cm(mmHg⋅sec)^(-1)^ ) | Capillary filtration coefficient | Healthy  Tumor | 3.6×10^-8^  2.8×10^-7^ | [[20](#_ENREF_20)]  [[21](#_ENREF_21)] |
| S_c_ /V_c_  (cm^-1^) | Capillary surface area per volume | Healthy  Tumor | $70$  200 | [[22](#_ENREF_22)]  [[23](#_ENREF_23)] |
| p_c_ (mmHg) | Capillary blood pressure (hydrostatic) | All | $15.6$ | [[24](#_ENREF_24)] |
| p_L_ (mmHg) | Lymphatic pressure (hydrostatic) | All | 0  0 |  |
| θ_L_  (mmHg s)^-1^ | Lymphatic filtration coefficient | Healthy  Tumor | 0.05  ~0 | [[25](#_ENREF_25)] |
| π_c_ (mmHg) | Plasma oncotic pressure | All | 20 | [[24](#_ENREF_24)] |
| π_i_ (mmHg) | Interstitial oncotic pressure | Healthy  Tumor | 10  15 | [[26](#_ENREF_26)]  [[21](#_ENREF_21)] |
| σ̅ | Osmotic reflection coefficient | Healthy  Tumor | 0.91  0.82 | [[27](#_ENREF_27)]  [[21](#_ENREF_21)] |

*Notes*: These parameters govern fluid exchange. We set blood pressure p_c_~15.6 mmHg for both (an average between arteriolar and venular ends). Healthy tissue has functioning lymphatics (θ_L_ moderately low); tumor tissue is assumed to effectively lack lymphatic drainage (θ_L_~0). The oncotic pressures are standard (plasma ~20–25 mmHg) and interstitium (~10 mmHg in healthy tissue). Tumors often have elevated interstitial protein content; we used 15 mmHg for the tumor interstitial oncotic pressure. The reflection coefficient σ̅ (which ranges from 0–1) is lower in tumors, reflecting leakier capillaries that allow more plasma proteins to escape.

Table S6: Initial conditions in the simulations (pretreatment).

| parameter | Description | Tissue | Value | Ref |
| --- | --- | --- | --- | --- |
| IFP_0_ (mmHg) | Interstitial pressure | Healthy  Tumor | 0  12 | [[28](#_ENREF_28)] |
| (C_O2_)_0_ (mol/m^3^) | O_2_ concentration in tissue | Healthy  Tumor | 0.02850  0.02424 | [[2](#_ENREF_2)] [[3](#_ENREF_3)] (calc.) |
| n_0_ (cells/cm^3^) | Cell density | Healthy  Tumor | 0  191 | - |

*Notes*: The breast tumor region was initialized as a partially developed tumor (moderate cell density, elevated but not maximal IFP, and mild hypoxia). Healthy tissue starts with normal physiological conditions (no excess IFP, ambient oxygen ~40 mmHg O₂, equivalent to 0.0285 mol/m³ O₂ based on solubility).

Using published measurements, we further computed the initial oxygen levels in benign and malignant breast tissue. Specifically, benign (normal) breast tissue exhibits ~83.8% oxygen saturation, whereas malignant (tumor) tissue has ~71.3% [[2](#_ENREF_2)]. Assuming a mean blood volume concentration of ~34 μM (0.034 mol/m³) in breast tissue [[3](#_ENREF_3)], this yields an oxygen concentration of ~0.02850 mol/m³ O₂ for benign tissue and ~0.02424 mol/m³ for malignant tissue. We used these values as the initial tissue oxygen concentrations in our model.

# S3. Boundary conditions

1. **Boundary conditions:** As described in the Methods, we applied the following boundary conditions in the model:

- **Mechanical:** On the outer boundaries of the breast tissue domain (far from the tumor), all displacement components were fixed at zero (u = 0). This simulates the restraint of surrounding tissue/body structures and prevents rigid-body motion.The interface between tumor and normal tissue had continuity of displacements and stresses (automatically satisfied in COMSOL by default, since it’s an internal boundary between two elastic domains).
- **Fluid (Darcy) and species:** The outer boundaries of the domain were set to no-flux for interstitial fluid, oxygen, and cells. This means ***v****_f_​ ⋅* ***n*** *= 0* (no fluid leaves the boundary) and *∇C_O2_​​​ ⋅* ***n*** *= 0, ∇n​​​ ⋅* ***n*** *= 0* (zero normal gradients for oxygen and cell density), where ***n*** is the outward normal. These conditions reflect an impermeable enclosure – reasonable if the domain boundary is taken sufficiently far such that very little flow or diffusion reaches it. In practice, our domain was large enough that the tumor’s influence did not reach the outer boundary significantly.

# References (supplementary)

1. Wagner BA, Venkataraman S, Buettner GR. The rate of oxygen utilization by cells. Free Radical Biology and Medicine. 2011;51(3):700-12.

2. Huang Z, Tian H, Luo H, Yang K, Chen J, Li G, et al. Assessment of Oxygen Saturation in Breast Lesions Using Photoacoustic Imaging: Correlation With Benign and Malignant Disease. Clinical Breast Cancer. 2024;24(4):e210-e8. e1.

3. Durduran T, Choe R, Culver J, Zubkov L, Holboke M, Giammarco J, et al. Bulk optical properties of healthy female breast tissue. Physics in medicine & biology. 2002;47(16):2847.

4. Narod S. Disappearing breast cancers. Multidisciplinary Digital Publishing Institute; 2012. p. 59-60.

5. Hafner M, Niepel M, Chung M, Sorger PK. Growth rate inhibition metrics correct for confounders in measuring sensitivity to cancer drugs. Nature methods. 2016;13(6):521-7.

6. Powathil G, Kohandel M, Milosevic M, Sivaloganathan S. Modeling the spatial distribution of chronic tumor hypoxia: implications for experimental and clinical studies. Computational and Mathematical Methods in Medicine. 2012;2012.

7. Swabb EA, Wei J, Gullino PM. Diffusion and convection in normal and neoplastic tissues. Cancer research. 1974;34(10):2814-22.

8. Burgess PK, Kulesa PM, Murray JD, Alvord Jr EC. The interaction of growth rates and diffusion coefficients in a three-dimensional mathematical model of gliomas. Journal of Neuropathology & Experimental Neurology. 1997;56(6):704-13.

9. DrugBank. Losartan: Uses, Interactions, Mechanism of Action [Available from: <https://go.drugbank.com/drugs/DB00678>.

10. Wilson CB, Lammertsma AA, McKenzie CG, Sikora K, Jones T. Measurements of blood flow and exchanging water space in breast tumors using positron emission tomography: a rapid and noninvasive dynamic method. Cancer research. 1992;52(6):1592-7.

11. Yazdannejat H, Hosseinimehr S, Ghasemi A, Pourfallah T, Rafiei A. Losartan sensitizes selectively prostate cancer cell to ionizing radiation. Cellular and Molecular Biology. 2016;62(1):30-3.

12. Liu W-B, Wang X-P, Wu K, Zhang R-L. Effects of angiotensin II receptor antagonist, Losartan on the apoptosis, proliferation and migration of the human pancreatic stellate cells. World Journal of Gastroenterology: WJG. 2005;11(41):6489.

13. Umemoto T, Ueno E, Matsumura T, Yamakawa M, Bando H, Mitake T, et al. Ex vivo and in vivo assessment of the non-linearity of elasticity properties of breast tissues for quantitative strain elastography. Ultrasound in Medicine & Biology. 2014;40(8):1755-68.

14. Samani A, Zubovits J, Plewes D. Elastic moduli of normal and pathological human breast tissues: an inversion-technique-based investigation of 169 samples. Physics in Medicine & Biology. 2007;52(6):1565.

15. Angeli S, Stylianopoulos T. Biphasic modeling of brain tumor biomechanics and response to radiation treatment. Journal of Biomechanics. 2016;49(9):1524-31.

16. Ramazanilar M, Mojra A. Characterization of breast tissue permeability for detection of vascular breast tumors: An in vitro study. Materials Science and Engineering: C. 2020;107:110222.

17. Moulavi A, Yazdani M. Monte Carlo simulation of two 106Ru eye plaques in a new mathematical human eye model. 2008.

18. Yao W, Li Y, Ding G. Interstitial fluid flow: the mechanical environment of cells and foundation of meridians. Evidence-Based Complementary and Alternative Medicine. 2012;2012.

19. Peng X, Janićijević Ž, Lemm S, Hauser S, Knobel M, Pietzsch J, et al. Impact of viscosity on human hepatoma spheroids in soft core–shell microcapsules. Advanced Healthcare Materials. 2024;13(11):2302609.

20. Rippe B, Kamiya A, Folkow B. Simultaneous measurements of capillary diffusion and filtration exchange during shifts in filtration‐absorption and at graded alterations in the capillary permeability surface area product (PS). Acta Physiologica Scandinavica. 1978;104(3):318-36.

21. Baxter LT, Jain RK. Transport of fluid and macromolecules in tumors. I. Role of interstitial pressure and convection. Microvascular Research. 1989;37(1):77-104.

22. Pappenheimer JR, Renkin E, Borrero L. Filtration, diffusion and molecular sieving through peripheral capillary membranes: a contribution to the pore theory of capillary permeability. American Journal of Physiology-Legacy Content. 1951;167(1):13-46.

23. Hilmas DE, Gillette EL. Morphometric analyses of the microvasculature of tumors during growth and after x‐irradiation. Cancer. 1974;33(1):103-10.

24. Brace RA, Guyton AC. Interaction of transcapillary Starling forces in the isolated dog forelimb. American Journal of Physiology-Heart and Circulatory Physiology. 1977;233(1):H136-H40.

25. Voutouri C, Stylianopoulos T. Evolution of osmotic pressure in solid tumors. Journal of Biomechanics. 2014;47(14):3441-7.

26. Wiederhielm CA. Dynamics of capillary fluid exchange: a nonlinear computer simulation. Microvascular Research. 1979;18(1):48-82.

27. Ballard K, Perl W. Osmotic reflection coefficients of canine subcutaneous adipose tissue endothelium. Microvascular Research. 1978;16(2):224-36.

28. Nathanson SD, Nelson L. Interstitial fluid pressure in breast cancer, benign breast conditions, and breast parenchyma. Annals of surgical oncology. 1994;1:333-8.
