# Supplementary material for: Losartan as a mechanotherapeutic adjuvant: Remodeling the breast tumor microenvironment to improve treatment efficacy
Source: PLoS One. 2025 Jul 11;20(7):e0328196. doi: 10.1371/journal.pone.0328196 (PMC12250197; doi:10.1371/journal.pone.0328196)
Supplement: Attachment — Submitted filename: Manuscript Review_ Losartan.docx [file pone.0328196.s002.docx]

Manuscript Number: PONE-D-25-24060

Article Type: Research Article

Full Title: Losartan as a mechanotherapeutic adjuvant: remodeling the breast tumor

microenvironment to improve treatment efficacy

Short Title: Losartan as a mechanotherapeutic adjuvant for breast tumor microenvironment

remodeling

Reviewer comments:

Although this study is highly theoretical, it demonstrates strong biological relevance, offers novel insights into tumor microenvironment modulation, and is a valuable contribution worthy of publication.

Dr Ang Soo Fan

Medical Oncologist

MBBS (UM) MRCP (UK) Medical Oncology (Singapore)
