# Supplementary material for: Losartan as a mechanotherapeutic adjuvant: Remodeling the breast tumor microenvironment to improve treatment efficacy
Source: PLoS One. 2025 Jul 11;20(7):e0328196. doi: 10.1371/journal.pone.0328196 (PMC12250197; doi:10.1371/journal.pone.0328196)
Supplement: Attachment — Submitted filename: Response to Reviewers.docx [file pone.0328196.s003.docx]

**Response to Reviewers**

**Manuscript ID:** PONE-D-25-24060
**Title:** *Losartan as a Mechanotherapeutic Adjuvant: Remodeling the Breast Tumor Microenvironment to Improve Treatment Efficacy*

We sincerely thank the editor and the reviewers for their thoughtful and constructive comments. We have carefully addressed all comments and revised the manuscript accordingly. Below, we provide our detailed point-by-point responses.

**Reviewer #1:**

Although this study is highly theoretical, it demonstrates strong biological relevance, offers novel insights into tumor microenvironment modulation, and is a valuable contribution worthy of publication.

**Response:**

We sincerely thank the reviewer for this encouraging and positive evaluation of our work.

**Reviewer #2:**

1. **Although the Laws of Physics apply universally, effects resulting from these Laws may be different in macro vs micro scales.**

**Response:**

We thank the reviewer for this valuable comment. Our original manuscript already acknowledges this limitation in the Discussion. Specifically, we have stated that:

“The framework operates at a single spatial scale (macroscale) and does not incorporate multi-scale (cellular-to-tissue level) interactions, thus potentially missing important cross-scale dynamics.”

2. **Mathematical modeling may not reflect actual observable results in Clinical Trials, in relation to emphasis placed on Deterministic Vs Stochastic modeling.**

**Response:**

In the original manuscript, we acknowledged that by stating:

“In addition, many model parameters (e.g., tissue stiffness, drug efficacy) were estimated from preclinical studies due to the limited availability of patient-specific data, which may introduce uncertainty and reduce predictive accuracy.”

To further address the reviewer’s concern about deterministic versus stochastic modeling, we have expanded the Discussion section to explicitly recognize that:

“In addition, many model parameters (e.g., tissue stiffness, drug efficacy) were estimated from preclinical studies due to the limited availability of patient-specific data. Consequently, the deterministic nature of our computational model fails to capture the biological variability and patient heterogeneity observed in clinical settings, which may introduce uncertainty and reduce predictive accuracy.”

3. **Malignant Cellular adaptive mechanisms to survive when faced with treatment challenges may be evident during Clinical Trials.**

**Response:**

In the original manuscript, we did not explicitly consider tumor adaptations (such as **the development of drug resistance)** that could allow cancer cells to survive therapy. To address this point, we have added a statement to the Discussion, highlighting that our model does not include dynamic resistance mechanisms. We acknowledge that cancer cells in patients may adapt over time. Specifically, we have added the following statement to the Discussion section:

*“Our model* *also does not account for tumor adaptive resistance mechanisms (e.g., genetic or phenotypic changes that enable cancer cells to survive therapy). As a result, it may overestimate treatment efficacy, since in clinical settings, cancer cells can adapt and develop resistance during prolonged therapy.”*

4. **The population of subjects selected for Clinical Trials may need to be of a certain target group, because of time constraints in relation to the disease progression versus the time needed for the contributory effects of Losartan to take effect.**

**Response:**

We thank the reviewer for highlighting this important point about the timing of losartan’s effects in relation to disease progression. In response, we have added a statement to the Discussion section to address this clinical consideration. Specifically, we have added the following statement to the Discussion section:

*“Because losartan’s stromal normalization effects develop gradually over several weeks, patients with more aggressive or rapidly progressing disease may not fully benefit if the tumor progresses before sufficient remodeling occurs.”*

5. **The Mathematical Model, which reflects local disease control and progression, may not have taken into consideration the importance of metastatic disease which may ultimately determine prognosis, and metastases as we know, occur quite early before the disease can be clinically detected.**

**Response:**

The original study focused only on the local tumor microenvironment and did not account for metastasis. We agree that this omission is a significant limitation. We have added text to the Discussion section explicitly acknowledging that our model is limited to the primary tumor and does not capture metastatic spread, which may critically influence outcomes. Specifically, we have added the following statement to the Discussion section:

*“Finally, the current model focuses only on the primary tumor and does not account for metastasis, even though metastatic spread often occurs early, sometimes even before the primary tumor becomes clinically detectable.”*
